# Inhibitors of Deubiquitinating Enzymes Interfere with the SARS-CoV-2 Papain-like Protease and Block Virus Replication In Vitro

**DOI:** 10.3390/v14071404

**Published:** 2022-06-27

**Authors:** Maximilian Große, Christian Setz, Pia Rauch, Janina Auth, Martina Morokutti-Kurz, Vladimir Temchura, Ulrich Schubert

**Affiliations:** 1 Institute of Virology, Friedrich–Alexander University Erlangen–Nürnberg (FAU), 91054 Erlangen, Germany; maximilian.grosse@uk-erlangen.de (M.G.); christian.setz@uk-erlangen.de (C.S.); pia.rauch@uk-erlangen.de (P.R.); janina.auth@fau.de (J.A.); vladimir.temchura@fau.de (V.T.); 2 Marinomed Biotech AG, A-2100 Korneuburg, Austria; martina.morokutti-kurz@marinomed.com

**Keywords:** COVID-19, SARS-CoV-2, coronavirus, deubiquitinating enzymes, papain-like protease, ubiquitin proteasome system, USP7, inhibitors of deubiquitinating enzymes

## Abstract

The ubiquitin proteasome system (UPS), particularly its deubiquitinating enzymes (DUBs), play a key role in the replication cycle of coronaviruses. The SARS-CoV-2 papain-like protease (Plpro) is known to process the viral polyproteins to form the replicase transcriptase complex and to counteract the host viral response. Recently, it was shown that this viral protease can also act as a deubiquitinating enzyme. In this study, we demonstrate that certain DUB-Inhibitors (DIs) interfere with SARS-CoV-2 replication. The DIs PR-619 and HBX41108 restrict SARS-CoV-2 in both Vero B4 and human Calu-3 lung cells where cells were infected with a Multiplicity of Infection (MOI) of 0.02. An in vitro protease assay using recombinant Plpro and Amido-4-methylcoumarin (AMC)-conjugated substrate revealed that PR-619 and HBX41108 are able to block the protease at concentrations where the interventions restricted virus replication. In contrast, DIs that do not inhibit Plpro had no influence on virus replication, which indicated that the protease might be at least one major target. Future vertical studies that would gain more insights into the mechanisms of how DUBs effect the replication of SARS-CoV-2 will further validate them as a potential therapeutic target.

## 1. Introduction

The COVID-19 pandemic, caused by the emergence of the Severe Acute Respiratory Syndrome Coronavirus type-2 (SARS-CoV-2), has resulted in around 514 million global cases and 6.2 million global deaths [1]. Despite vaccination campaigns, the emergence and spread of SARS-CoV-2 variants remain a major threat to public health. These “Variants of Concern” (VOCs) are the result of viral mutability and have the potential to evade the vaccine- or infection-induced antiviral immune response [2,3]. By now, five SARS-CoV-2 variants are classified as VOCs by the World Health Organization (WHO): SARS-CoV-2 Alpha [4] (also referred to as B.1.1.7 [5]), Beta [6] (also referred to as B.1.351 [5]), Gamma [7] (also referred to as P.1 [5]), Delta [8] (also referred to as B.1.617.2 [5]) and Omicron (also referred to as B.1.1.529 [9]), which is currently predominant. Assuming the emergence of future variants, the development of effective therapeutic and broadly acting countermeasures remains as of utmost importance.

SARS-CoV-2 belongs to the genus betacoronaviruses and is closely related to SARS-CoV-1, which caused an outbreak of atypical pneumonia in 2002–2003 [10]. Both interact with the human angiotensin converting enzyme 2 receptor (hACE2) present on several host cells, the binding of which is mediated by highly glycosylated spike proteins [11,12,13]. Upon binding, SARS-CoV-2 can enter the cell either via membrane fusion after cleavage of the spike glycoprotein by the host cell protease TMPRSS2 [14] or, alternatively, via endocytosis facilitated by endosomal proteases such as cathepsin L [15,16].

Regarding current therapeutic treatment options for patients with COVID-19, large randomized studies such as the RECOVERY and the WHO Solidarity trials showed therapeutic benefit only for low dose treatment with dexamethasone [17], while other repurposed drugs such as Hydroxychloroquine (HCQN) [18] or Remdesivir (RDV) [19,20,21] were controversially discussed. However, in contrast to HCQN, some clinical trials revealed a benefit for RDV, which led to the approval of this repurposed drug for treatment of COVID-19 [20,21]. There are currently approved monoclonal antibodies for high-risk patients, which need to be administered at an early time point after infection [22]. Moreover, interleukin receptor blockers, janus kinase inhibitors and glucocorticoids were administered to COVID-19-patients and specific antiviral small molecules were approved [23]. Nirmatrelvir, an inhibitor of the 3-Chymotrypsin-like protease (3CLpro) of SARS-CoV-2 combined with Ritonavir, a small drug originally developed as a Human Immunodeficiency Virus (HIV)-1-protease inhibitor, was developed by Pfizer. The drug is approved in combinatory use as Paxlovid^®^ for high-risk COVID-19 patients [22,24]. Moreover, Molnupiravir, targeting the RNA-dependent RNA-Polymerase from SARS-CoV-2, received an emergency approval for high-risk COVID-19 patients in some countries, e.g., USA and Japan, but not in the EU [25].

Hitherto, several vaccines have been authorised from the European Medicines Agency (EMA), including two mRNA vaccines from Pfizer–BioNTech and Moderna as well as three vaccines based on viral vectors from Johnson & Johnson, AstraZeneca and Novavax [26,27]. However, herd-immunity might be difficult to achieve, as the vaccines do not confer complete immunity [28]. There is ongoing concern that SARS-CoV-2 will transform into an endemic virus causing seasonal severe respiratory infections. All this highlights the unmet urgent need to develop prophylactic as well as therapeutic agents that are safe, widely available and broadly acting against different viral strains.

As SARS-CoV-2 shares 79.5% sequence homology with SARS-CoV-1, the antiviral research also focuses on drugs that have been proven effective against SARS-CoV-1 [29]. In addition to directly acting antiviral drugs, prone for developing drug resistances, particularly against viruses with a high evolution rate, broadly active and drug ir-resistant antivirals that mainly target cellular factors have been of growing interest in pharmaceutical developments. Among them are inhibitors of the ubiquitin proteasome system (UPS), which have also been shown to restrict the replication of coronaviruses [30].

Cellular protein degradation in the cytosol of eukaryotes is primarily mediated by the ATP-dependent UPS, where targeted substrates are tagged with multimers of ubiquitin for subsequent degradation by the 26S proteasome [31]. To target a protein into the UPS, the attachment of at least four ubiquitin moeties is necessary. However, this process can be reversed by so-called deubiquitinating enzymes (DUBs) [32,33]. Generally, these enzymes play several roles in cellular processes, such as maintainance of free ubiquitin, as well as regulation of protein functions. More than 90 DUBs have been discovered so far and they can be divided into five families: cysteine proteases; ubiquitin specific protease family (USP); ubiquitin C-terminal hydrolases (UCHs); ovarian tumor proteases (OTS); and the Josephine family, as well as zinc metallo-proteases JAB1-MPN-Mov34 (JAMMs) [34,35]. In addition to cellular DUBs, a number of viral DUBs have been identified, which are used to evade host immune response and boost viral replication [36]. Specific DUB-Inhibitors (DIs) are now available and offer the opportunity to selectively modulate DUB activities with a relatively low range of toxicity and off-target activities [37].

It has been pointed out that the UPS, and in particular DUBs, play a major role in various viral infection cycles, both pro- and antiviral. Viruses have the ability to either encode their own proteins with DUB function or modify cellular proteins of the UPS for their advantage [38,39]. An essential step in the replication of SARS-CoV-2 is the autocatalytic processing of the replicase polyproteins into 16 nonstructural proteins (NSPs) by the viral papain-like protease (Plpro) and the 3Clpro, of which some assemble into the viral replication–transcription complex. Central components of this complex are NSP12 (RNA-dependent RNA polymerase (RdRp)) as well as the cofactors NSP7 and NSP8. It has been shown previously that the Plpro of SARS-CoV-1 exhibits deubiquinating activity [30,40,41]. As Plpro of SARS-CoV-1 shares 82.9% sequence identity with the same protease of SARS-CoV-2 [42], it was legitimate to hypothesize that DIs might interfere with SARS-CoV-2 replication [43]. Indeed, we demonstrate here that the DIs PR-619 and HBX41108 effectively block SARS-CoV-2 replication, without affecting cell viability. Moreover, these DIs are able to interfere with the Plpro at a similar dose range, where the DIs restricted virus replication in vitro, indicating that the inhibition of Plpro represents one possible mechanism for the antiviral activity of those DIs.

## 2. Materials and Methods

### 2.1. Viruses

The virus strain SARS-CoV-2_PR-1_ was isolated from a 61-year-old patient and amplified in Vero B4 cells as described previously [44]. Viral titers were determined by an endpoint titration assay. For the generation of new virus stock, a T75 cell culture flask of Vero B4 cells was infected with the isolate. Virus-containing cell culture supernatant was harvested at 72 h postinfection (hpi), centrifuged and passed through a 0.45 µm pore-size filter. Cells were split twice per week for up to 10 passages. Virus stocks were stored at −80 °C until further usage. 

For Multiplicity of Infection (MOI) determination of SARS-CoV-2_PR-1_, Vero B4 cells were infected with serial dilutions of the virus stock for 72 h. Afterward, cells were fixed (4% PFA), permeabilized (0.5% Triton/PBS), blocked (1% BSA/PBS-T) and finally stained with a SARS-CoV-2 nucleoprotein (NP) antibody (Biozol, Eching, Germany). The endpoint of virus infection was analyzed via immunofluorescence microscopy (as described in Section 2.7), and viral titer was calculated by the method of Reed and Muench [45].

### 2.2. Cell Culture

Vero B4 and 293T cells were maintained in Dulbecco’s Modified Eagle’s Medium (DMEM) containing 10% (*v*/*v*) inactivated fetal calf serum (FCS), 2 mM l-glutamine, 100 U/mL penicillin and 100 µg/mL streptomycin. 

Calu-3 (human lung adenocarcinoma) cells were cultured at 37 °C with 5% CO_2_ in MEM containing 20% FCS, with 2 mM l-glutamine, 100 U/mL penicillin, 100 µg/mL streptomycin and 2 mM sodium pyruvate. Furthermore, 293T-ACE2 cells were produced as described in [46] and maintained in DMEM containing 10% FCS, with 2 mM l-glutamine.

### 2.3. Determination of Viral RNA Copies from Released Viruses by qRT-PCR

Virus–containing samples were quantified by real-time PCR Luna Universal Probe One-Step RT-PCR Kit from New England Biolabs (Cat: E3006L, Ipswich, MA, USA) allowing reverse transcription, cDNA synthesis and PCR amplification in a single step. Samples were analyzed by 7500 software v2.3 (Applied Biosystems, Waltham, MA, USA). PCR primers were used according to [47]: RdRp_fwd: 5′-GTG-ARA-TGG-TCA-TGT-GTG-GCG-G-3′ and RdRp_rev 5′-CAR-ATG-TTA-AAS-ACA-CTA-TTA-GCA-TA-C-3′. Probe was 5′--CAG-GTG-GAA-/ZEN/CCT-CAT-CAG-GAG-ATG-C -3′ (Label: FAM/IBFQ Iowa Black FQ). A dsDNA-polynucleotide sequence (Integrated DNA Technologies, Coralville, IA, USA) was used as a positive control: 5′-TAA-TAC-GAC-TCA-CTA-TAG-GGT-ATT-GAG-TGA-AAT-GGT-CAT-GTG-TGG-CGG-TTC-ACT-ATA-TGT-TAA-ACC-AGG-TGG-AAC-CTC-ATC-AGG-AGA-TGC-CAC-AAC-TGC-TTA-TGC-TAA-TAG-TGT-TTT-TAA-CAT-TTG-GAA-GAG-ACA-GGT-ACG-TTA-ATA-GTT-AAT-AGC-GTA-CTT-CTT-TTT-CTT-GCT-TTC-GTG-GTA-TTC-TTG-CTA-GTT-ACA-CTA-GCC-ATC-CTT-ACT-GCG-CTT-CGA-TTG-TGT-GCG-TAC-TGC-TGC-AAT-ATT-GTT-3′. This polynucleotide sequence contains parts of the SARS-CoV-2 Envelope (E) and RdRp genes and was used as the standard for the determination of viral RNA copies in our experiments. Generating a series of dilutions (104, 105, 106 and 107 copies/mL) of this standard, the experiments were quantified by the use of a standard curve to obtain absolute values of RNA copies in the sample.

### 2.4. Inhibitors

PR-619 was purchased from Calbiochem (Darmstadt, Germany) and dissolved in DMSO resulting in a stock concentration of 10 mM. HBX41108, IU-1 and LDN-57444 were obtained from Sellekchem (Houston, TX, USA) and dissolved in DMSO, resulting in a stock solution of either 5 mM, 50 mM or 10 mM, respectively. RDV was obtained from Cayman Chemical (Ann Arbor, MI, USA) and dissolved in DMSO, resulting in a stock solution of 1 mM. Camostat mesylate was purchased from Cayman Chemical (Ann Arbor, MI, USA) and dissolved in DMSO, resulting in a stock solution of 10 mM. The SARS-CoV-2 Spike-specific antibody Tres-V618 was obtained from CoVER antibodies GmbH (Bubenreuth, Germany). All interventions were used at the concentrations indicated in the different experiments. 

### 2.5. Infection Experiments

For Western Blot and qRT-PCR analysis, 1 × 10^5^ cells/well Vero B4 were seeded in 24-well plates, and following the formation of monolayers, cells were infected with the field isolate SARS-CoV-2_PR-1_ at an MOI of 0.02 in FCS-free DMEM. At 1 h postinfection, the input virus was removed, and cells were treated with interventions. At 72 hpi, supernatants were harvested and released virions were purified through a 20% (*w*/*v*) sucrose cushion (20,000× *g*, 4 °C, 90 min) and analyzed via Western blot or incubated for 10 min at 95 °C and finally used for qRT-PCR analysis.

For immunofluorescence microscopy analysis, a total of 1 × 10^4^ Calu-3 cells/well were seeded in 96-well plates and infected with SARS-CoV-2_PR-1_ with an MOI of 0.02 the next day. After 1 h of infection, the cells were treated with inhibitors for 30 h. To this end, supernatants were harvested and analyzed via qRT-PCR as described above, while cells were fixed with 4% PFA and further analyzed by immunofluorescence microscopy.

### 2.6. SDS-PAGE and Western Blotting

Protein samples generated by infection experiments were separated by SDS-PAGE, transferred onto nitrocellulose membranes, blocked with 3% bovine serum albumin and incubated with the appropriate primary antibody (Ab). Viral proteins were detected by antibodies derived from convalescent SARS-CoV-2 patient sera. The antihuman and antirabbit secondary antibodies coupled to horseradish peroxidase (HRP) were obtained from Dianova (Hamburg, Germany).

### 2.7. Immunofluorescence Microscopy

For immunostaining, the cells were fixed with 4% PFA for 15 min. Following a washing step, cells were blocked and permeabilized overnight with 1% BSA in PBS (+0.2% Triton) and, afterwards, stained with a polyclonal rb anti-NP antibody (GeneTex, GTX135357, Irvine, CA, USA) for 24 h. Furthermore, plates were incubated for 1 h with a goat anti-rb-AlexaFlour488 (Invitrogen, A11008, Waltham, MA, USA) and, finally, stained with DAPI for 10 min. For immunostaining, the fluorescence output of Alexa488 was quantitatively analyzed with a Perkin Elmer VictorX4 (488 nm) and pictures were taken using a CTL- ELISPOTreader. 

### 2.8. Protease Activity Measurement In Vitro

In order to measure the activity of the SARS CoV-2 protease, recombinant Plpro (10 nM; R&D Biosystems, #E-611-050, Minneapolis, MN, USA) was mixed with the Interferon-stimulated gene 15 (ISG-15)-Amido-4-methylcoumarin (AMC; R&D Biosystems, #UL-553-050, Minneapolis, MN, USA) substrate (800 nmol) in a 96-well plate with a black bottom. Shortly after, protease activity was determined for 40 min using a Perkin Elmer VictorX4 (excitation: 380 nm; emission: 460 nm). In case of treatment with interventions, Plpro was pretreated for 1 h. 

### 2.9. Assessment of Cell Viability

Viability of infected and treated cells was assessed by the water-soluble tetrazolium salt (WST)-1 assay (Cat.: 5015944001, Roche, Penzberg, Germany) according to the manufacturer’s instructions.

### 2.10. Production of Lentiviral Pseudoparticles Expressing SARS-CoV-2 Spike Protein and Luciferase

Pseudoparticles were produced according to [48]. Next, 2 × 10^7^ 293T cells were seeded in a T175 flask. After 24 h, the cells were transiently transfected with pGEA-LucW, pADSIV3+ and pCG-SARS-CoV-2-Alpha using polyethyleneimine (PEI). Then, 2 days post-transfection, the cell culture supernatants were harvested, centrifuged, passed through a 0.45 µm pore size filter and, afterwards, stored at −80 °C until further usage. 

### 2.11. Lentiviral Vector-Based SARS-CoV-2 Neutralization Assay

The neutralization assay was conducted in accordance with [48]. Then, 1 × 10^5^ 293T-ACE2 cells/well were seeded in 96-well plates one day prior to infection. Particles were pretreated with interventions for 1 h at 37 °C. Afterwards cells were infected with 1:2.5 dilutions of pretreated particles of SARS-CoV-2 Spike pseudotyped lentivirus (Luc reporter). At 48 h after infection, plates were incubated for 3 min with luciferase reagent (One Glow, Promega, Madison, WI, USA) and, afterwards, luciferase activity was measured using a Perkin Elmer VictorX4 reader. 

### 2.12. Software and Statistics

We used Microsoft Word and Excel. GraphPad Prism 9.0 was used for statistical analyses and to generate graphs. Figures were generated with CorelDrawX7. Other software used included Gen5 v.3.04, Perkin Elmer 2030 and X for measurement and analysis of fluorescence data. To evaluate the results obtained by qRT-PCR, 7500 software v2.3 was used. 

## 3. Results

### 3.1. Treatment with DIs Restrict Viral Replication of SARS-CoV-2 in Vero B4 Cells 

**Figure 1:**
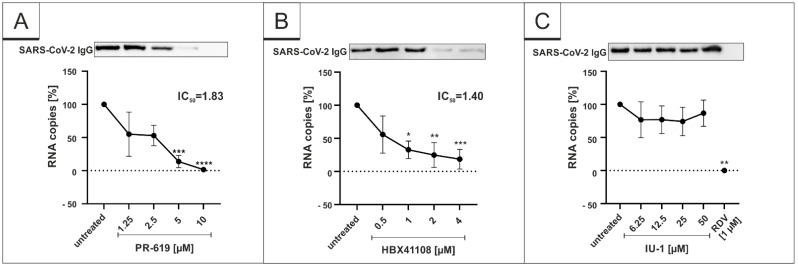
PR-619 and HBX41108 inhibit SARS-CoV-2 replication in Vero B4 cells. qRT-PCR of cell culture supernatants and Western blot analysis of released virions after treatment with (**A**) PR-619, (**B**) HBX41108 and (**C**) IU1. Remdesivir (RDV) was used as a positive control (**C**). One representative Western blot is shown. Cell culture supernatants were harvested 3 days postinfection (dpi). The virions were purified and analyzed by Western blot using a SARS-CoV-2 convalescent serum. Bars show mean values of five (**A**,**B**) or three (**C**) independent experiments ± standard deviation. Statistical analysis was performed using a multiple comparison Kruskal–Wallis test (Anova) followed by Dunn’s post hoc test (* *p* <0.05; ** *p* < 0.01; *** *p* <0.001).

To reveal whether DIs affect the replication of SARS-CoV-2 in vitro, we analyzed the release of viral proteins, as well as RNA copies from infected cultures with and without DI treatment. Therefore, Vero B4 cells were infected with SARS-CoV-2_PR-1_ and further treated with the broadly active DI PR-619, which was described to inhibit USP7 and USP47, the DUBs Josephin domain containing 2 (JOSD2), deneddylase 1 (DEN1), UCH-L3, UCH-L5, USP2, 4, 5, 8, 14, 15, 20 and 28 [49]. Western blot analysis of the virus fractions showed a significant decrease in the accumulation of proteins after treatment with PR-619 in a dose-dependent manner (Figure 1A). This is in concert with the results obtained by qRT-PCR, which demonstrate a similar dose-dependent reduction in RNA copies released in the cell culture medium after treatment with PR-619 (Figure 1A). 

Next, we investigated the restriction capability of more specific DIs, namely, the USP7-inhibitor HBX41108 and the USP14-inhibitor IU-1. Those DIs were purposely chosen as the tertiary structure of SARS-CoV-1 Plpro, which has DUB activity by itself and is known to be similar to the DUBs USP7 and 14 [41]. Interestingly, HBX41108 was able to inhibit the viral replication in SARS-CoV-2-infected Vero B4 cells in a similar way (Figure 1B) as PR-619 (Figure 1A), whereas the USP14-specific inhibitor IU-1 showed no effect (Figure 1C). The IC_50_ values differed slightly. While PR-619 exerted its antiviral activity with an IC_50_ value of 1.83 µM (Figure 1A), the IC_50_ value for HBX41108 was 1.40 µM (Figure 1B). In one infection experiment, RDV was enrolled as positive control at 1 µM, which blocked the SARS-CoV-2 replication completely (Figure 1C). 

**Figure 2:**
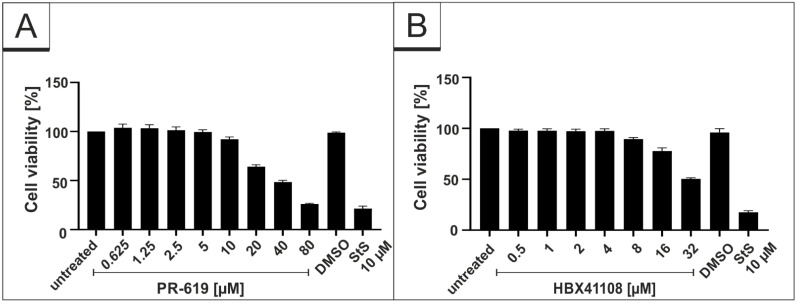
Influence of PR-619 and HBX41108 on cell viability in Vero B4 cells. Following treatment with (**A**) PR-619 and (**B**) HBX41108 for three days, the cell viability was measured by water-soluble tetrazolium salt (WST)-1 assays. Bars represent mean values of three independent experiments ± SD. Staurosporine (StS).

To control for potential unspecific effects of drug treatment on cell viability, water-soluble tetrazolium salt (WST)-1 assays were performed in uninfected Vero B4 cells. Treatment at concentrations, which were able to inhibit SARS-CoV-2 replication, had no impact on cell survival for all interventions (Figure 2). IU-1 also had no toxic effect at the concentrations, which were used in the infection experiments (data not shown). 

### 3.2. Treatment with DIs Restrict Viral Replication of SARS-CoV-2 in Calu-3 Cells

**Figure 3:**
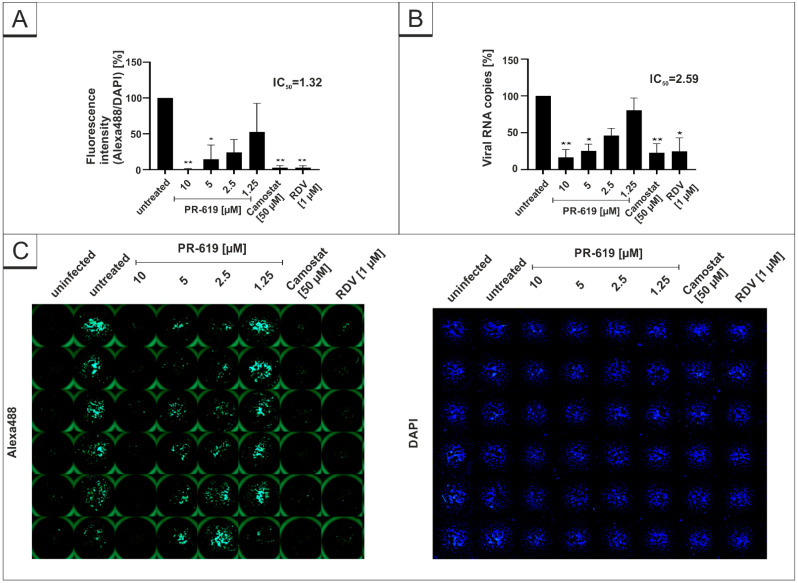
PR-619 inhibits SARS-CoV-2 replication in Calu-3 cells. Quantitative analysis of fluorescence microscopy (**A**), qRT-PCR of cell culture supernatants (**B**) and representative images of fluorescence staining (**C**) 30 h post infection. Cells were analyzed via microscopy using anti-SARS-CoV-2 NP-antibody. Bars show mean values of three independent experiments ± standard deviation. Statistical analysis was performed using a multiple comparison Kruskal–Wallis test (Anova) followed by Dunn’s post hoc test (* *p* < 0.05; ** *p* < 0.01).

As our results indicate that certain DIs have the potential for antiviral candidates, we aimed to investigate if the DIs, which showed an antiviral effect in Vero B4 cells, exhibit similar antiviral properties in human Calu-3 cells, representing the most extensively studied surrogate lung cell-infection model. Therefore, cells were infected with the wildtype isolate SARS-CoV-2_PR-1_ at the same MOI used before (Figure 1) and subsequently treated with different concentrations of the DIs PR-619 and HBX41108 for 30 h. Cell culture supernatants were harvested as described above and analyzed via qRT-PCR. In addition, cells were fixed and further stained with SARS-CoV-2-specific antibodies for evaluation of intracellular expression of the SARS-CoV-2 nucleoprotein (NP). RDV, as well as Camostat mesylate, were used as a positive control at 1 µM or 50 µM, respectively. Both interventions effectively suppressed SARS-CoV-2 replication (Figure 3). Similar to the results observed in Vero B4 cells, PR-619 shows a dose-dependent antiviral effect against SARS-CoV-2 in human Calu-3 lung cells (Figure 3). 

**Figure 4:**
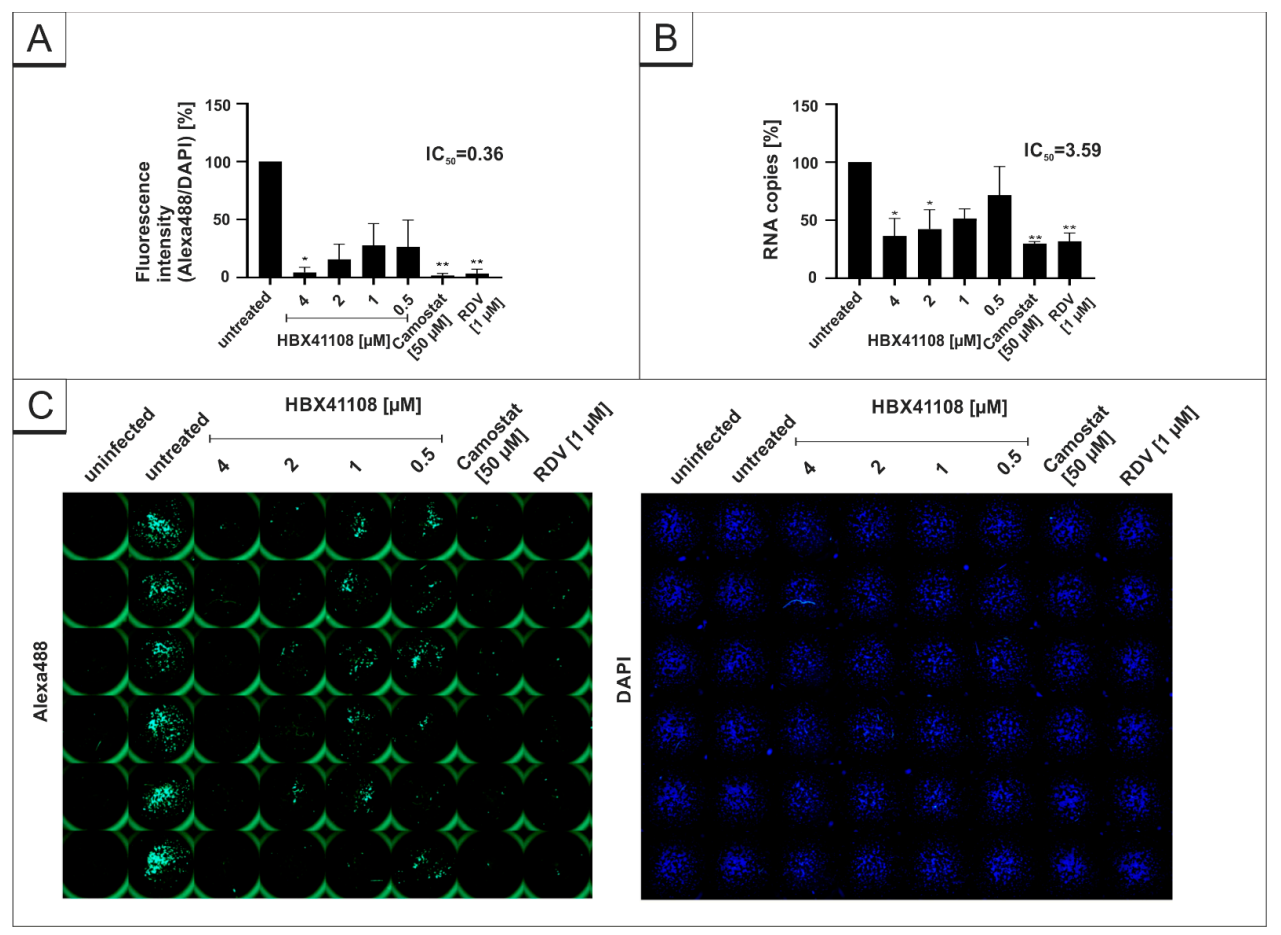
HBX41108 inhibits SARS-CoV-2 replication in Calu-3 cells. Quantitative analysis of fluorescence microscopy (**A**), qRT-PCR of cell culture supernatants (**B**) and representative images of fluorescence staining (**C**) 30 h post infection. Cells were analyzed via microscopy using anti-SARS-CoV-2 NP-antibody. Bars show mean values of three independent experiments ± standard deviation. Statistical analysis was performed using a multiple comparison Kruskal–Wallis test (Anova) followed by Dunn’s post hoc test (* *p* < 0.05; ** *p* < 0.01).

Furthermore, the same experiments were conducted with the DI HBX41108. In concert with the results obtained in Figure 1, HBX41108 shows a dose-dependent antiviral effect in human Calu-3 lung cells (Figure 4). The IC_50_ values differed slightly between the different experiments (Figure 4A,B). Using microscopic analysis, an IC_50_ of <0.5 µM was established (Figure 4A), whereas for qRT-PCR, the IC_50_ value was ~3.59 µM (Figure 4B). 

**Figure 5:**
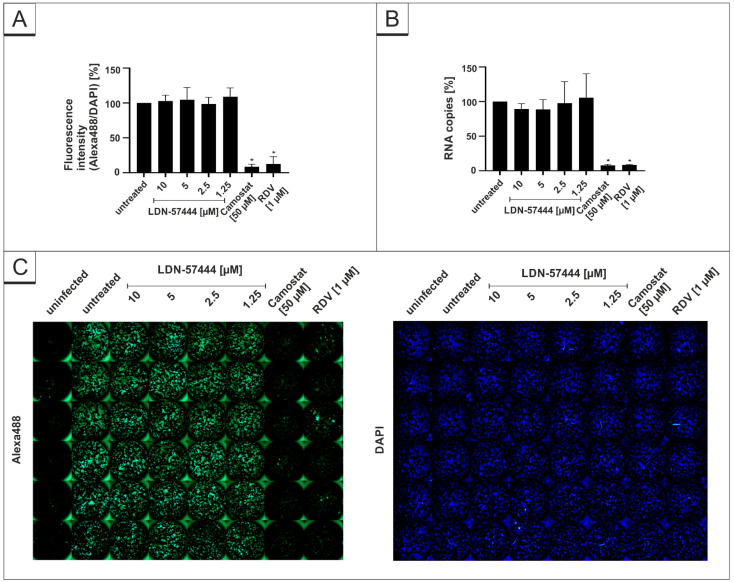
LDN-54777 does not inhibit SARS-CoV-2 replication in Calu-3 cells. Quantitative analysis of fluorescence microscopy (**A**), qRT-PCR of cell culture supernatants (**B**) and representative images of fluorescence staining (**C**) 30 h post infection. Cells were analyzed via microscopy using anti-SARS-CoV-2 NP-antibody. Bars show mean values of three independent experiments ± standard deviation. Statistical analysis was performed using a multiple comparison Kruskal–Wallis test (Anova) followed by Dunn’s post hoc test (* *p* < 0.05).

In order to control for the specificity of the used DIs, we further investigated the antiviral effect of the DI LDN-57444, which inhibits UCH-L1 and 3, but neither USP7 nor USP14. Using the same experimental setup, LDN-57444 does not exert any antiviral effects against SARS-CoV-2, whereas the positive controls Camostat mesylate and RDV deployed full antiviral activity (Figure 5). 

**Figure 6:**
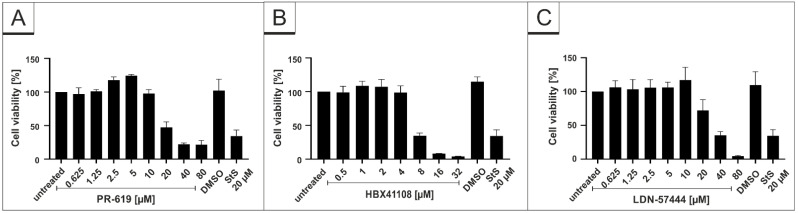
Influence of PR-619, HBX41108 and LDN-57444 on cell viability in Calu-3 cells. Following treatment with (**A**) PR-619, (**B**) HBX41108 and (**C**) LDN-57444 for 30 h, the cell viability was measured by water-soluble tetrazolium salt (WST)-1 assays. Bars represent mean values of three independent experiments ± SD. Staurosporine (StS).

The DAPI staining in each experiment revealed no toxic effect of the substances at the used concentrations (Figure 3C, Figure 4C and Figure 5C). Additionally, WST-1 assays were performed in uninfected Calu-3 under identical conditions as for the virus infection experiments. The results summarized in Figure 6 demonstrate that treatment with all DIs at concentrations which effectively suppress SARS-CoV-2 replication exhibit no impact on cell viability in Calu-3 cells (Figure 6). Staurosporin was used as a positive control at a concentration of 20 µM. To control for unspecific toxic effects of the solvent itself, DMSO was added in the same amount as to the highest used amount of each intervention (80 µM PR-619, 32 µM HBX41108 and 80 µM LDN-57444). 

### 3.3. HBX41108 Shows No Antiviral Effect When Used in a Lentivirus-Based Pseudoviral Assay

**Figure 7:**
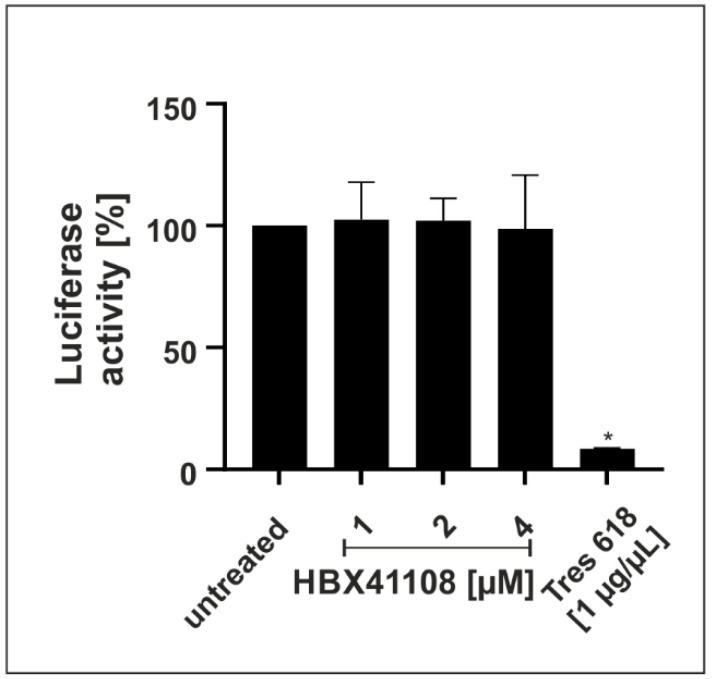
Effect of HBX41108 on SARS-CoV-2-spike-driven entry. Quantitative analysis of Luciferase activity following infection of 293T-ACE2 with Alpha-spiked lentiviral pseudoparticles. Particles were treated with interventions prior to infection for 1 h. The efficiency of infection in cell lysates was determined by measuring the luciferase activity 48 h post infection. Bars represent mean values of three independent experiments ± SD. Statistical analysis was performed using a multiple comparison Kruskal–Wallis test (Anova) followed by Dunn’s post hoc test (* *p* < 0.05).

Infection experiments in Vero B4 and Calu-3 cells demonstrated a strong antiviral effect of the DI HBX41108. Next, we wanted to investigate if the intervention exerts its antiviral activity by interfering with spike-mediated binding and thereby affecting the entry of the virus. Therefore, a SARS-CoV-2 pseudovirus neutralization assay, based on a replication-incompetent lentivirus expressing an Alpha SARS-CoV-2 S protein, was performed. The spike-specific antibody Tres-V618 was used as a positive control (Figure 7). Treatment with HBX41108 up to concentrations of 4 µM, which completely blocked the replication of SARS-CoV-2 in infection experiments with DIs (Figure 1B and Figure 4), showed no effect (Figure 7), suggesting that the intervention does not affect the viral entry, but rather acts on later steps of the viral replication cycle. 

To control for potential unspecific effects of drug treatment on cell viability in the pseudoviral assay, water-soluble tetrazolium salt WST-1 assays were performed in uninfected 293T-ACE2 cells 48 h after treatment. Concentrations of the interventions, which were used in the SARS-CoV-2 pseudoviral assay, had no impact on cell viability (data not shown).

### 3.4. PR-619 and HBX41108 Inhibit the Protease Activity of SARS-CoV-2 Plpro

**Figure 8:**
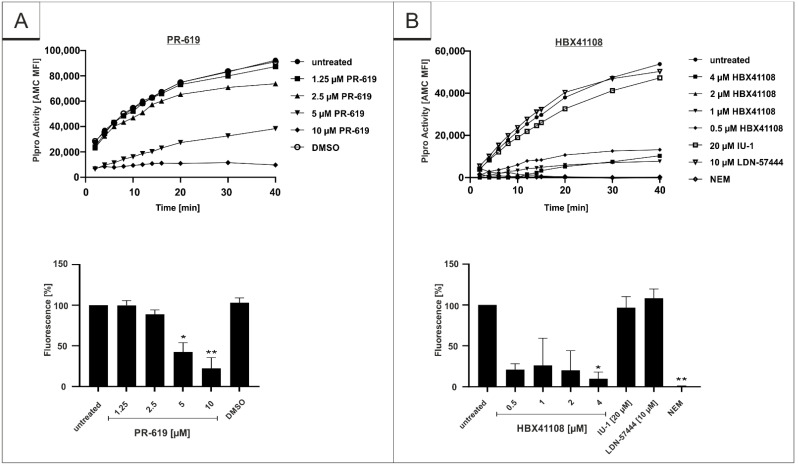
Influence of (**A**) PR-619 and (**B**) HBX41108, IU-1 and LDN-57444 on proteolytic activity of SARS-CoV-2 papain-like protease. After pretreatment of the protease with interventions for 1 h, the proteolytic activity was measured with ISG15-Amido-4-methylcoumarin. Protease activity was determined for 40 min using a Victor-Reader (excitation: 380 nm; emission: 460 nm) and further quantified by calculation of the Area under the curve (AUC). Bars represent means of three independent experiments ± SD. Additionally, one representative enzyme kinetic is shown (upper part of the figure). N-Ethylmaleimide (NEM) was used as a positive control at a concentration of 10 mM. Statistical analysis was performed using a multiple comparison Kruskal–Wallis test (Anova) followed by Dunn’s post hoc test (* *p* < 0.05; ** *p* < 0.01).

Recently, it was shown that Plpro can act as a viral deubiquinating enzyme [30,40,41]. It was reported that the inhibition of the virally encoded DUB, Plpro, interferes with the replication of the SARS-CoV-1 [50]. Since HBX41108 shows no antiviral effect when used in a viruslike particle (VLP)-derived virus entry-assay, it was intriguing to hypothesize that the DI potentially interacts with SARS-CoV-2 directly by interfering with the protease activity of Plpro, as the tertiary structure of SARS-CoV Plpro is known to be similar to the DUB USP7 [41]. To evaluate our hypothesis, a protease assay on the basis of the Plpro-specific substrate (ISG-15) was established. Recombinant SARS-CoV-2 Plpro was pretreated for 1 h with DIs PR-619 and HBX41108 that were effective in the infection assays and with DIs IU-1 and LDN-57444, which showed no effect. After the addition of AMC-conjugated ISG-15, protease activity was determined for 40 min. The results showed that both DIs, which exhibit antiviral activity against SARS-CoV-2, show a significant and dose-dependent inhibitory effect on the enzymatic activity of Plpro (Figure 8A,B). In contrast, IU-1 and LDN-57444, which showed no antiviral effect in SARS-CoV-2 infection experiments (Figure 1C and Figure 5), were not able to interfere with the protease activity (Figure 8B). N-Ethylmaleinimid (NEM), an alkylating ir-reversible inhibitor of all cysteine proteases, was used as positive control and blocked SARS-CoV-2 Plpro in the protease assay completely (Figure 8B). 

## 4. Discussion

Since the beginning of the SARS-CoV-2 outbreak, the pandemic led to global public health issues. In view of newly emerging variants and viruses, and the need for a pandemic preparedness against those, there is still a tremendous need for antiviral therapeutics against SARS-CoV-2. Viral proteases are one of the most obvious potential targets for drug development. In the case of SARS-CoV-2, Pfizer developed a drug combination of Nirmatrelvir, an inhibitor of the 3CLpro of SARS-CoV-2 [51], with Ritonavir, a small drug that inhibits the HIV-1 protease [52]. This product received emergency-approval by the FDA in December 2021 and was found to reduce the risk of hospitalization or death by 89% compared to placebo [53]. The approach to block SARS-CoV-2 by a combinatory drug treatment has also successfully been shown in several other studies in vitro [54,55,56,57]. 

Our data suggest that the antiviral effect of PR-619 and HBX41108 is potentially due to the inhibition of the protease Plpro (Figure 8). This might pave the way for the development of DIs as directly acting antiviral agents. Both SARS-CoV-2 proteases, Plpro and 3CLpro, are necessary to process the polyproteins of SARS-CoV-2 into the different NSPs, of which several are crucial for the replication of the virus. While Plpro cleaves NSP1-3, 3CLpro cleaves NSPs 4-16 [41,58]. As these proteases process different parts of the SARS-CoV-2 NSPs, a combinatory treatment of DIs with Paxlovid^®^ or other 3CLpro inhibitors could be an advantage to completely block the replication of SARS-CoV-2 by an additive or even synergistic effect. By inhibiting both proteases, a large part of the replication machinery of the virus would be restricted. 

DUBs, as part of the UPS, are important for the viral replication of coronaviruses, and the Plpro of SARS-CoV-1 and SARS-CoV-2 acts as a DUB itself [30,40,43]. The Plpro of SARS-CoV-1 not only processes viral precursor proteins, but also acts as viral enzyme cleaving post-translational Ub-moieties from target proteins to evade antiviral immune responses [30,40,41,43,59]. While Plpro of SARS-CoV-1 cleaves ubiquitin as well as the ubiquitin-like protein ISG-15, the protease of SARS-CoV-2 predominantly targets ISG-15 [43]. SARS-CoV-1 shares 79.5% sequence homology with the recently emerged SARS-CoV-2 [29]. The inhibition of SARS-CoV-1 Plpro by DIs was shown previously by Ratia et al. using a Western blot approach that measured the processing of viral proteins [50]. By using a colorimetric in vitro protease assay, we now demonstrate that the DIs PR-619 and HBX41108 inhibit the enzymatic function of SARS-CoV-2 Plpro (Figure 8), a mode of action that potentially contribute to their antiviral activity (Figure 1, Figure 3 and Figure 4).

The protease from SARS-CoV-2 contains different enzymatic activities: (i) processing of the viral polyprotein 1a (pp1a) by recognizing the consensus cleavage sequence LxGG, which results in the formation of the nonstructural proteins 1–3 [60,61]; and (ii) deconjugation of the two-domain Ub-like protein interferon-stimulated gene 15 (ISG-15) [43,62]. The tertiary structure of SARS-CoV-1 Plpro is known to be similar to the DUBs USP7 and 14 [41]. The deISGylating activity of Plpro might be blocked by the addition of PR-619 and HBX41108, as both inhibitors target the DUB USP7, which shares structural similarities with the Plpro [41]. In addition, it has been shown that SARS-CoV-2 Plpro does not only play a role in the processing of viral proteins and virus proliferation, but also has major effects on innate immunity [43]. The protease cleaves cellular ISG-15 moieties from post-translational modified proteins and thereby protects SARS-CoV-2 against the antiviral immune responses of the host [43]. The inhibition of ISG-15 processing by Plpro might have an influence on the innate immune response in vivo. Following type I interferon stimulation, ISG-15 is activated and further regulates the immune response by interferon-γ (IFN-γ) and the cytokine production. Thereby, ISG-15 mediates protection against a variety of viruses, amongst them Influenza A and B, Hepatitis B and C, HIV-1 and HPV-16 [63,64]. However, viruses have evolved countermeasures to antagonize ISG-15 and thus escape the innate immune response [63]. SARS-CoV-2 Plpro antagonistic activity against ISG-15 might block the production of various cytokines involved in the activation of the innate immune response against viral infection, e.g., Type I IFN-β and chemokines such as CXCL10 and CCL5 [41,43]. Recently, it was shown that Plpro-mediated reduction of IRF3 ISGylation, a known key player in antiviral immune responses, can be reversed by specific Plpro inhibitors [43,65]. Hence, by using PR-619 and HBX41108, the innate immunity against SARS-CoV-2 might be clearly enhanced in vivo.

Previously, we demonstrated that PR-619 increases the polyubiquitination of HIV-1 Gag protein and thus its entry into the UPS and the MHC class I pathway [66]. By design, modern mRNA vaccines drive transient expression of protein antigens accessible to MHC class I processing machinery. Since CD8+ T cells are important effector cells that expand in the early protection window after prime SARS-CoV-2 spike mRNA vaccination [67] and maintain recognition of variants of concern due to epitope conservation [68], it might also be beneficial to coadministrate the DIs together with SARS-CoV-2-spike mRNA vaccines.

However, the reduction in virus replication following treatment with PR-619 and HBX41108 could also be explained by the direct inhibition of the cellular DUB USP7. The protein is abundant in the nucleus and has proviral properties, as several Herpes viruses bind to USP7 to restrict its interaction with cellular proteins [69,70,71]. Furthermore, USP7 deubiquitinates the HIV Tat protein leading to an enhanced HIV-1 production [72]. This is in concert with the results obtained from the infection experiments (Figure 1, Figure 3 and Figure 4) and the protease assay (Figure 8). As the IC_50_ values of PR-619 and HBX41108 are lower in the infection experiments (Figure 1, Figure 3 and Figure 4) than their inhibitory effect in the protease assay (Figure 8), it can be assumed that the reduction of protease activity is not the only mechanism by which DIs exert their antiviral effect. Hence, SARS-CoV-2 may utilize USP7 or other DUBs for its replication, and the used DIs exert their antiviral activity not only by interfering with the Plpro but also by directly targeting cellular DUBs.

DUB-Inhibitors were originally developed for treatment of cancer or neurodegenerative diseases [73], and several studies showed promising results in different cell lines and, most importantly, also in animal studies [74]. Some studies showed that DIs do not only exert anticancer effects, but also have potential against viral infections [75]. The DI HBX was shown to restrict the replication of human Adenovirus Typ 5 (hAdV5) [76]. Furthermore, antiviral activity of DIs against several RNA viruses was proposed [77], e.g., against Dengue virus [78] or SARS-CoV-1 [50]. Preclinical studies with various DIs were initiated, demonstrating their potential in research, especially in the area of oncology [79]. However, as many DIs target not only one DUB, the development of specific compounds that target exclusively one, specific DUB is of utmost importance. Several side effects of the DIs, analyzed in vivo, are an ongoing problem and most likely represent one reason why promising clinical trials for the treatment of cancer were terminated [80]. Although our results indicate no toxic effects of our DIs in vitro (Figure 2 and Figure 6), the clinical use of these compounds as antiviral agents requires the standard preclinical evaluation prior to any first-in-man study.

## Data Availability

Data are included in the article.
